# Evaluation of Hydrogels Based on Oxidized Hyaluronic Acid for Bioprinting

**DOI:** 10.3390/gels4040082

**Published:** 2018-10-09

**Authors:** Matthias Weis, Junwen Shan, Matthias Kuhlmann, Tomasz Jungst, Jörg Tessmar, Jürgen Groll

**Affiliations:** Department for Functional Materials in Medicine and Dentistry, University of Würzburg, Pleicherwall 2, 97070 Würzburg, Germany; mannie.weis@googlemail.com (M.W.); junwen.shan@fmz.uni-wuerzburg.de (J.S.); Matthias.Kuhlmann@coltene.com (M.K.); Tomasz.juengst@fmz.uni-wuerzburg.de (T.J.); joerg.tessmar@fmz.uni-wuerzburg.de (J.T.)

**Keywords:** biofabrication, bioprinting, hyaluronic acid

## Abstract

In this study, we evaluate hydrogels based on oxidized hyaluronic acid, cross-linked with adipic acid dihydrazide, for their suitability as bioinks for 3D bioprinting. Aldehyde containing hyaluronic acid (AHA) is synthesized and cross-linked via Schiff Base chemistry with bifunctional adipic acid dihydrazide (ADH) to form a mechanically stable hydrogel with good printability. Mechanical and rheological properties of the printed and casted hydrogels are tunable depending on the concentrations of AHA and ADH cross-linkers.

## 1. Introduction

Hydrogels based on polysaccharides have attracted a high level of attention in recent decades, especially in the fields of Tissue Engineering and Regenerative Medicine [1,2,3,4]. The polysaccharides used for these applications are biopolymers, which are chosen in order to mimic the structural support of the native extracellular matrix and allow the three dimensional growth and proliferation of incorporated cells [5,6]. Hyaluronic acid, as one prominent example, is a polysaccharide which is an exisiting component of different extracellular matrices and therefore quite promising as cell carrier for several in-vitro approaches [7]. However, in many cases the natural polysaccharides need to be specially functionalized using chemically active groups for cross-linking reactions to improve the mechanical stability and ensure the shape fidelity of the newly formed hydrogel constructs [8].

In our group, thiol-ene chemistry has been used successfully to develop a hydrogel system based on thiol-functionalized hyaluronic acid together with allyl-functionalized poly(glycidol)s, which can be cast as a cell carrier hydrogel and bioprinted, allowing a generation of better defined 3D hydrogel constructs [9]. The group of Boccaccini introduced an oxidized alginate/gelatin-based hydrogel system for bioprinting. The aldehyde functionalities of the oxidized alginate were used for secondary cross-linking with the amino functions of gelatin using Schiff Base chemistry [10]. Without subsequent reduction, the Schiff Base chemistry provides a reversible reaction of an aldehyde with an amine, followed by elimination of water to form the final imine, which is however still labile to hydrolysis. Special amine derivatives, such as hydrazide or aminooxy groups, lead to much greater hydrolytic stability via formation of hydrazones or oximes [11], which might be interesting for the long term application of the hydrogels. The hydrazone system, as one example, has been published recently by the group of Burdick, which used an excess of adipic acid dihydrazide (ADH) to covalently functionalize acid functions of hyaluronic acid (HA) using carbodiimide chemistry as multiple hydrazide species and combined this polymer with oxidized HA (AHA) as aldehyde species [12]. With this strategy, they created two multifunctional and macromolecular cross-linking partners for a stable but still reversible hydrogel. Su et al. also cross-linked AHA with ADH, forming an injectable hydrogel for nucleus pulposus regeneration [13].

In this study, we investigated oxidized hyaluronic acid (AHA), synthesized from low (LMW) and high molecular weight (HMW) HA with different oxidation degrees, for its hydrogel formation as well as suitability for bioprinting with ADH as low molecular weight bifunctional cross-linker. Since the Schiff Base system using both hydrazide functions of the small ADH leads to a chemically cross-linked network in dynamic equilibrium, we expected tunable printability of the obtained polysaccharide hydrogels by varying the cross-linker concentration.

## 2. Results and Discussion

### 2.1. AHA Synthesis and Characterization

**Figure 1:**
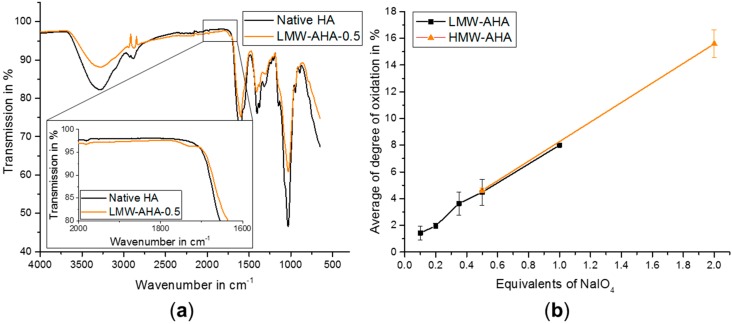
(**a**) IR-spectra of native hyaluronic acid (HA) (black) and low molecular weight oxidized hyaluronic acid (LMW-AHA-0.5) (orange) with a zoom to the shoulder at 1730 cm^−1^; (**b**) Average of degree of oxidation (DO) according to equivalents of NaIO_4_ used, referred to one dimer unit of HA.

The oxidation of HA was performed using NaIO_4_ introducing dialdehyde functionalities in several HA dimer units, resulting in a simultaneous ring opening of the glucuronic acid [14]. Aldehyde formation was proven by Fourier-transform infrared (FTIR) spectroscopy with the apparent presence of a shoulder at 1730 cm^−1^ beside the fingerprint area (Figure 1a) [13]. Quantification of the aldehyde groups was performed using a 3-methyl-2-benzothiazolinone hydrazone hydrochloride assay (MBTH-assay). As expected, the degree of oxidation (DO) increased with increasing amounts of oxidation reagent used for reaction. By varying the amounts of the oxidation reagent, we achieved a DO between 1.4% and 8.0% for LMW-AHA and 4.6% and 15.6% for HMW-AHA (Figure 1b), which proves good control over the amount of available aldehyde functions.

### 2.2. Rheology

**Figure 2:**
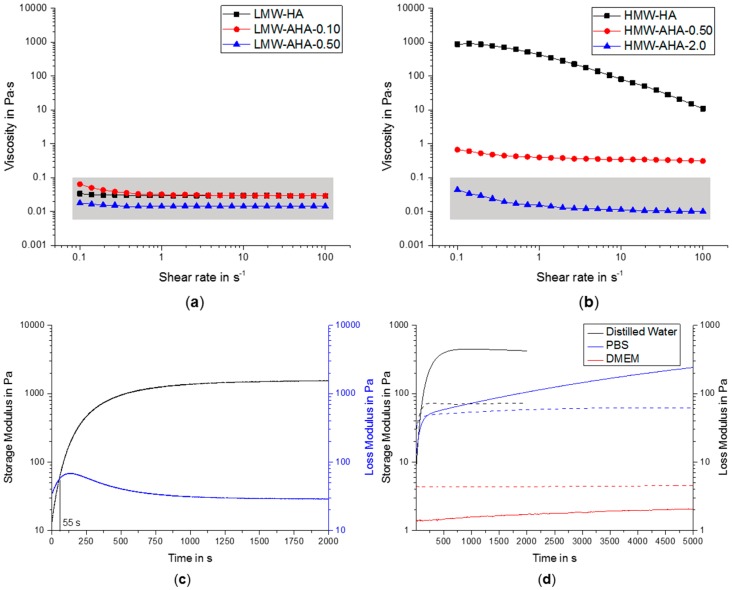
(**a**) Viscosities of LMW-HA and LMW-AHA with lowest and second highest equivalents of NaIO_4_, determined at increasing shear stress; (**b**) Viscosities of HMW-HA and HMW-AHA with lowest and highest equivalents of NaIO_4_, determined at increasing shear stress; (**c**) Determination of the gelation point of gel with 3.5% (*w*/*w*) HMW-AHA-0.5 and 0.10% (*w*/*w*) adipic acid dihydrazide using time sweep measurements; (**d**) Determination of gelation kinetics (storage (continuous line) and loss (dashed lines) modulus) of identical gel compositions with 3.5% (*w*/*w*) HMW-AHA-0.5 and 0.20% (*w*/*w*) ADH mixed in different solvent (distilled water, PBS and DMEM).

The oxidation of HA, however, was accompanied with a significant decrease in viscosity of the pure AHA solutions (5% *w*/*w*), indicating a progressing degradation of HA chains during oxidation. Depending on the amount of NaIO_4_ used, viscosity of LMW-AHA varied between 0.01 to 0.06 Pa∙s, and higher amounts of oxidizing reagent tended to result in lower viscosity and consequently stronger degradation (Figure 2a). This was observable for both LMW-AHA and HMW-AHA (Figure 2b), whereas the highest oxidized HMW-AHA showed viscosity in a similar range as LMW-AHA.

The gelation and hydrogel formation of a representative sample of 3.5% (*w*/*w*) HMW-AHA-0.5 together with 0.10% (*w*/*w*) ADH cross-linker is shown in Figure 2c. For this combination, we determined a very short gelation time of less than 1 min, but it was still long enough to allow mixing of cells. In addition to the polymers in pure water, we also measured the gelation time of gels in other aqueous solutions. To improve cell compatibility, we compared demineralized water, phosphate buffered saline (PBS) and Dulbecco’s Modified Eagle Medium (DMEM) (Figure 2d). The gels in distilled water showed the highest storage and loss modulus whereas gels formed in DMEM had the lowest values. Additionally, the gel in water exhibited the shortest gelation time, while it did not gel at all in DMEM. Since DMEM contains several amino acids with free amine functions, the formation of Schiff Base to hydrazone may be massively disturbed by trimmed aldehydes via imine formations with the amino acids instead of the cross-linker ADH [15].

### 2.3. Mechanical Properties

**Figure 3:**
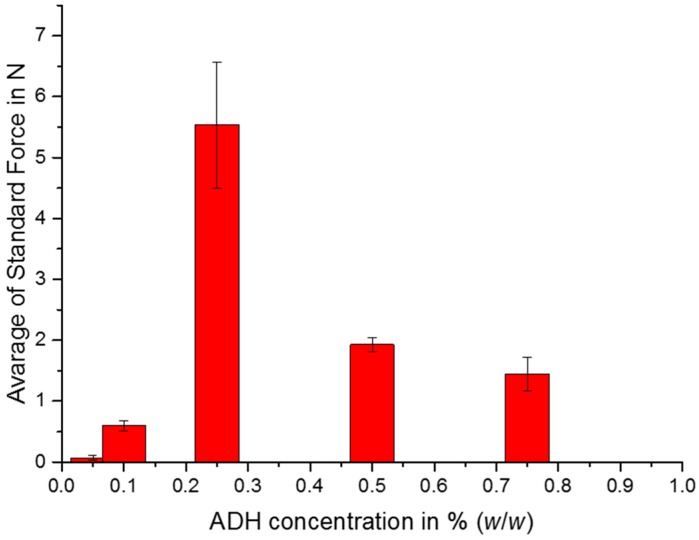
Average standard force for the confined compression measurements of gels containing 3.5% (*w*/*w*) of HMW-AHA-2.0 and various concentrations of ADH. Graph represents average of 4 individual samples and their standard deviation.

Confined compression tests were performed to compare cast gels with HMW-AHA-2.0 containing various concentrations of cross-linkers, since not all gels could be fully recovered from the 96-well-plates without destruction. In Figure 3, representative samples are shown and best compression stability was achieved with 0.25% (*w*/*w*) ADH. With increasing concentrations of the cross-linker, gels lose mechanical stability, since the aldehyde functionalities of AHA are saturated with ADH and are not able to form further stable cross-links between polymer chains.

### 2.4. Swelling Studies

**Figure 4:**
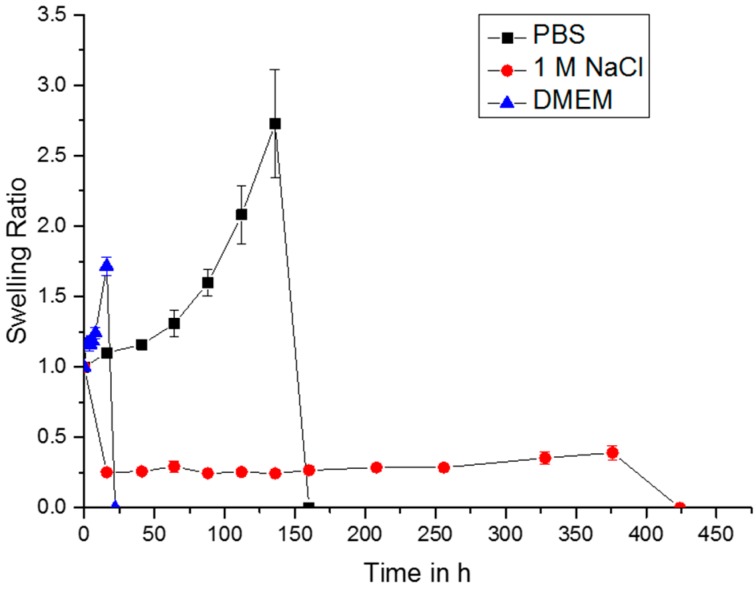
Swelling tests with gels in different solution media, containing 3.5% (*w*/*w*) HMW-AHA-2.0 and 0.5% (*w*/*w*) ADH.

Depending on the aqueous solutions the gels were incubated in, swelling ratios larger and smaller than 1 were obtained (Figure 4). Besides the different swelling studies shown in Figure 4, gels were also allowed to swell in pure distilled water. These data are not shown, since the gels were already dissolved overnight. Since the Schiff Base chemistry is reversible in water and a constant equilibrium of free and bound polymer chains is present, this effect leads to a permanent loss of polymer chains. Hyperosmolar solutions with high concentration of sodium ions tend to result in gel shrinkage and decelerate the degradation of the gels, since sodium ions also may have some shielding effects on the ionic repulsion between the negatively charged carboxylic groups of AHA. Accordingly, the gel in 1 M NaCl was the longest lasting up to 424 h, until it was not possible to weigh anymore. In DMEM and PBS, the hydrogels dissolved much faster, after two days for DMEM and six days for PBS. Both solutions, PBS and DMEM contain a plurality of salts and amino acids, which might interfere with the gel network. Especially with DMEM, amino acids will diffuse into the network of AHA and ADH, causing divergence of the polymer chains and subsequent gel breakdown. For PBS, which contains no amino acid, the degradation is most likely governed by the adjusted neutral pH, which is very likely to alter the reaction balance of the formed Schiff base.

### 2.5. Bioprinting

Based on determined viscosities of LMW-AHA and HMW-AHA and degrees of oxidation HMW-AHA-2.0 was chosen for the bioprinting experiments. Since HMW-AHA-2.0 shows similar viscosity as LMW-AHA after extensive degradation but has much higher degree of oxidation, many more aldehyde functions will be available for cross-linking. Additionally, ADH concentrations for cross-linking were varied and are listed in Table 1.

**Figure 5:**
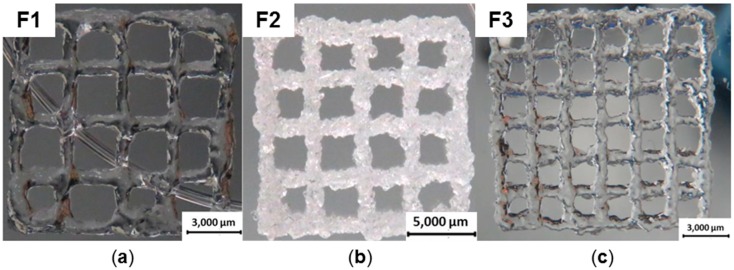
All gel compositions are listed in Table 1; (**a**) F1 was printed with 3 mm spacing between the strands; (**b**) F2 was printed with 3 mm spacing between the strands; (**c**) F3, printed with 2 mm spacing between the strands.

The gel composition used for formulation F1 was printed using a needle with a diameter of 0.25 mm at 3.4 bar. It exhibited the lowest shape fidelity and the strands began to lose their forms during the printing process (Figure 5a). After printing eight layers, the strands were no longer visible because of the low stability. The gel with highest stability was used for F2. Because of the immense stiffness, caused by higher concentration of ADH and consequent higher cross-linking density, it could only be printed with a very high pressure of 4.5 bar and a needle diameter of 0.41 mm, producing eight and twelve layers (Figure 5b) with a brittle appearance of the construct. The best printing results were achieved with F3 (Figure 5c). The pressure used for printing could be reduced to 2.0 bar and a needle diameter of 0.25 mm was applied. After eight layers, the construct showed smooth strands and shape fidelity. Additionally, a smaller distance between the strands was achieved compared to Wang et al. printing four layers with 3 mm between the strands [12].

## 3. Conclusions

Here we evaluated reversibly cross-linked hydrogels based on oxidized HA and ADH for 3D printing. Mechanical and swelling properties can vary greatly, depending on the gel composition. Three different gel compositions based on HMW-AHA-2.0 could be used for printing, since HMW-AHA-2.0 offers moderate viscosity and the highest aldehyde functionalization for cross-linking with various amounts of ADH. The short term stability of the formed hydrogels in PBS and DMEM is a beneficial feature for their use as sacrificial support materials, which can be easily washed out using cell compatible medium, e.g., DMEM. Still, the significant degradation of HA during the oxidation is a common issue, which must be solved for future studies by introducing aldehyde functionalities via other more gentle modifications. Alternatively, biocompatible reducing reagents can be considered for reducing the reversible hydrazone bond to a stable hydrazide species, which will increase stability under cell culture conditions and eventually allow long term applications as bioink. Moreover, the use of specially designed multifunctional macromolecular cross-linkers bearing several hydrazide functions might further enhance stability of the hydrogels. All in all, the hyaluronic acid based hydrogels presented here are a promising platform for future development of new bioinks based on modified polysaccharides.

## 4. Materials and Methods

### 4.1. Materials and Reagents

High (1.3 MDa) and low (50 kDa) molecular weight HA, abbreviated as HMW-HA and LMW-HA were purchased from Dagmar Kohler-BaccaraRose, Alpen, Germany. Adipic acid dihydrazide and 3-Methyl-2-benzothiazolinone hydrazone were acquired from Sigma-Aldrich, St. Louis, MO, USA. The following reagents were purchased from Merck KGaA, Darmstadt, Germany: Acetaldehyde, adipic acid dihydrazide (ADH), disodium phosphate dodecahydrate, potassium chloride, monopotassium phosphate, sodium chloride, sodium hydroxide, hydrochloride acid 32%. Calcium chloride dihydrate was acquired from Acros Organics, Geel, Belgium; Dulbecco’s Modified Eagle Medium (DMEM) from Invitrogen Life Technologies GmbH, Karlsruhe, Germany; iron(III) chloride from Alfa Aeser GmbH & Co. KG, Karlsruhe, Germany; ethylene glycol from Carl Roth GmbH & Co. KG, Karlsruhe, Germany; sodium periodate (NaIO_4_) from Thermo Fisher Scientific, Waltham, MA, USA; sulfamic acid from Grussing GmbH Analytica, Filsum, Germany.

### 4.2. Synthesis of AHA

The oxidation of HMW-HA and LMW-HA was performed according to Su et al., with some modifications. Throughout this manuscript, the products are termed according to the molecular weight of HA used and the equivalents of oxidizing reagent used for the oxidation step of the disaccharide unit. AHA is used as an abbreviation of aldehyde containing HA. For example, LMW-AHA-0.5 was obtained from the oxidation of low molecular weight HA and 0.5 eq. of NaIO_4_. Various amounts (0.1, 0.2, 0.35, 0.5 eq. for LMW-HA, 0.5 and 2.0 for HMW-HA) of NaIO_4_ referred to one dimer unit of HA were used to generate different degrees of oxidation. The reaction process of LMW-AHA-0.5 is described as a representative example. In short, 2 g of LMW-HA was dissolved in 100 mL distilled water. A solution of 0.586 g NaIO_4_ in 15 mL distilled water was added slowly and the mixture was stirred at room temperature for 2 h. To stop the reaction, 2 mL ethylene glycol was added and the reaction stirred for 1 h. For purification, the reaction mixture was dialyzed against distilled water for at least 3 days and lyophilized to obtain a white solid foam (Alpha 1–2 LD, Martin Christ Gefriertrocknungsanlage GmbH, Osterode, Germany). For synthesis with HMW-HA, the polysaccharide was dissolved in 160 mL instead of 100 mL to guarantee homogeneous solutions.

### 4.3. Characterization of AHA

FTIR-spectrometry (Nicolet iS10 FT-IR, Thermo Fisher Scientific, Waltham, MA, USA) was used to prove the formed aldehyde functions.

To determine the quantity of aldehyde functionalization, a MBTH-assay was performed. For this assay, a solution containing 1% (*w*/*w*) MBTH, a solution containing 1% (*w*/*w*) FeCl_3_, 1.6% (*w*/*w*) sulfamic acid and a solution with 0.01% (*w*/*w*) AHA were prepared. The MBTH solution was mixed with AHA solution in a ratio of 1:1 in a total volume of 400 µL. After 30 min 200 µL of the solution containing FeCl_3_ and sulfamic acid were added and chilled for another 10 min. The mixture was diluted to 1 mL and the absorption measured via UV/VIS-spectrometer (GENESYS 10 S Bio Spectrophotometer, Thermo Fisher Scientific, Waltham, MA, USA). Various concentrations (0.02, 0.05, 0.1, 0.2, 0.5, 1, 2, 5 and 10 µg/µL) of an acetaldehyde solution were treated using the same procedure for calibration.

### 4.4. Gelation with ADH

For cross-linking reactions, stock solutions of ADH (50 mg/mL), LMW-AHA (150 mg/mL) and HMW-AHA (50 mg/mL) in distilled water were mixed to form gels with final concentrations of 8.0, 9.0, 10.0, 11.0 and 12.0% (*w*/*w*) for LMW-AHA, 3.0, 3.5, 4.0 and 4.5% (*w*/*w*) for HMW-AHA and 0.050, 0.10, 0.25, 0.50, 0.75% (*w*/*w*) of ADH.

### 4.5. Rheology

Rheological measurements were carried out with a rheometer Physica MCR 301 from Anton Paar GmbH (Graz, Austria). A cone-plate setting with 60 mm cone diameter and an opening-angle of 0.5° was used for rotation measurements with shear rates between 0.1–100.0 s^−1^. Also, the gelation point of various hydrogel formations was investigated through taking oscillation measurements at 4 °C with a frequency of 15.9 s^−1^ and a deformation of 1.0%.

### 4.6. Mechanical Tests

Mechanical confined compression tests were performed with a universal testing machine (Zwick/Roell Z010 from Zwick GmbH & Co. KG, Ulm, Germany), using a 100-N load cell and maximum penetration depth of 2 mm. Gels were formed in 96-well-plates with a diameter of 6.4 mm and height of 6.2 mm.

### 4.7. Swelling Properties

For swelling properties, gels with a final concentration of 3.5% (*w*/*w*) of HMW-AHA-2.0 and 0.5% (*w*/*w*) ADH were tested in distilled water, phosphate buffered saline (PBS), 1.0 M NaCl solution and DMEM. The gels were incubated at 37 °C with the solutions for 16, 41, 64, 88, 112, 136, 160, 208, 256, 328, 376 and 424 h, and weighed after those times. The swelling ratio *q* was calculated with *m*_0_ (weight of gels before swelling) and *m_x_* (weight of gels at time *x*) via the following equation:

$$q=\frac{m_{0}}{m_{x}}$$

### 4.8. Bioprinting

Printing tests were performed with a bioplotter (3D Discovery Gen 1, RegenHU Ltd., Villaz-St-Pierre, Switzerland). Cannulas with a diameter of 0.25 and 0.41 mm and a pressure up to 4.5 bar were used for printing. For better adhesion of the printed constructs, the covers of microtiter plates were coated with a HA solution. The number of printed layers were 8 and 12. Gels with a final composition of 3.5% (*w*/*w*) HMW-AHA-2.0 with 0.05, 0.075 and 1.0% (*w*/*w*) ADH were used for printing.

## Figures and Tables

**Table 1:** Composition of gels and set ups used for bioprinting.

Specimen	HMW-AHA-2.0 Concentration in % (*w*/*w*)	ADH Concentration in % (*w*/*w*)	Needle Diameter in mm	Pressure in Bar
F1	3.5	0.05	0.25	3.4
F2	3.5	1.0	0.25	2.0
F3	3.5	0.075	0.41	4.5
